# Handbooks for Senior Students and Practitioners

**Published:** 1910-09-03

**Authors:** 


### THE MEDICAL LIBRARY.

### HANDBOOKS FOR SENIOR STUDENTS AND PRACTITIONERS.

Allbutt's "System of Medicine." 8 vols., 25s. each, Macmillan and Co. ("Allbutt" is deservedly ranked as one of the finest works on medicine, and is specially valuable because of the great amount of space given to consideration of treatment and pathology.)

Osler's "System of Medicine." Edited by Osler and McCrae. 7 vols, 24s. each volume. Oxford Medical Publications, 20 Warwick Square, E.C. (This system is one of the best which it is possible to obtain.)

Allchin's "Manual of Medicine." 5 vols., vols. i. to iv. 7s. 6d., vol. v. 10s. Macmillan and Co. (Shorter but very useful, and particularly good on diseases of special systems.)

Osler's "Practice of Medicine." 1 vol. Appleton. 21s. net. (An admirable text-book; well written and comprehensive.)

Roberts' "Medicine." 26s. 2 vols. H. K. Lewis, Gower Street. 16s. net.

Taylor's "Medicine." Churchill, 7 Great Marlborough Street. 16s. net. (A useful manual; exceedingly popular with students.)

Savill's "Medicine." 2 vols., 12s. 6d. each. Arnold.

Fagge and Pye Smith's "Medicine." 2 vols. 42s. net. Churchill.

Whitla's "Manual of Medicine." 2 vols. £1 12s. Henry Renshaw. (Sir Wm. Whitla's new work, in dictionary form; concise and practical.)

Carter's "Elements of Practical Medicine." 1 vol. 10s. 6d. H. K. Lewis. (A popular condensation, probably the best of its kind.)

Bury's "Clinical Medicine." 1 vol. 16s. Charles Griffin and Co., Exeter Street, London.

Monro's "Medicine." Baillière, Tindall, and Cox. 1 vol. 15s.

Mitchell Bruce's "Principles of Treatment." 1 vol. 15s. net. Oxford Medical Publications. (Another text-book now becoming a veteran. It is one of the most philosophic works to be found in British medical literature, and will certainly remain a standard work for many years to come.)

---

Spencer and Gask's "Theory and Practice of Surgery," 1 vol. 22s. net. Churchill. (Perhaps the best one-volume text-book obtainable.)

Rose and Carless' "Manual of Surgery," 1 vol. 21s. net. Baillère, Tindall, and Cox. (This text-book runs neck and neck with the preceding in popular favour.

Cheyne and Burghard's "Manual of Surgical Treatment," in six parts, 9s. to 18s. each vol. Longmans, Green. (Very complete, thoroughly practical, and in every way satisfactory.)

Thomson and Miles' "Manual of Surgery." 2 vols. 10s. 6d. each net. Oxford Medical Publications. (This text-book is a favourite one north of the Tweed, and deservedly.)

Jacobson's "Operations of Surgery," in two volumes. Churchill. 42s. net.

The most recent and sumptuous book on Operative Surgery is that edited by F. F. Burghard for the Oxford Medical Publications : 4 vols., 36s. each net. This work has only lately been published, and embodies the latest and most up-to-date methods of the distinguished surgeons who have contributed to it.

"A System of Surgery," edited by Sir Frederick Treves, in two volumes, 48s. Cassell and Co. (The work consists of an excellent series of articles by surgeons attached to the various London hospitals, without any attempt to make it encyclopædic. It may be supplemented by the new edition of Treves' "Manual of Operative Surgery," revised by the author and Mr. J. Hutchinson, jun., which is also published in two volumes by Messrs. Cassell, price 42s.)

Pearce Gould's "Elements of Surgical Diagnosis" is another old favourite with students; it occupies a position from which no more recent work seems capable of ousting it. (Cassell and Co., 9s.).

*Surgical Anatomy and Surgical Pathology* form the backbone of surgery as a science. But too many students are content to learn only so much as is sufficient to enable them to obtain a minimum examination knowledge. There are several good English text-books on surgical anatomy. One of the best is M'Lachlan's "Applied Anatomy : Surgical, Medical, and Operative," in two volumes, published at Edinburgh by Messrs. E. and S. Livingstone. It is clear, well written, and well illustrated.

Rawling's "Landmarks and Surface Markings of the Human Body," published by H. K. Lewis, 5s. net, is somewhat on the lines of the late Mr. Holden's "Medical and Surgical Landmarks." It is well illustrated.)

Sir Frederick Treves' "Surgical Applied Anatomy" has long been a favourite with students. A revised edition has been issued by Dr. Arthur Keith, and published by Messrs. Cassell and Co. It is in one volume, well illustrated.

Box and McAdam Eccles' "Clinical Applied Anatomy" (Churchill, 12s. 6d. net) is in many respects an interesting and useful volume.

Bowlby's "Surgical Pathology" (Churchill, 10s. 6d. net) contains a large number of drawings, and describes in simple language the various pathological processes with which the student of surgery should be acquainted. It is a useful and popular handbook.

Galabin's "Manual of Midwifery" (Churchill, 14s.). A thoroughly popular and useful handbook, practical and concise, just re-edited. Dakin's "Handbook of Midwifery" (18s.) is another favourite with students and practitioners. A new edition of this work is in course of preparation. Eden's "Manual of Midwifery" (Churchill, 12s. 6d. net) is perhaps the best small book for students.

Whitridge Williams' "Text-book of Obstetrics." An excellent work, well illustrated, by an American obstetrician.

Herman's "Difficult Labour" (Cassell, 12s. 6d.). An indispensable work. Equally admirable is Herman's "Diseases of Women" (Cassell, 1 vol. 25s.), the standard work on gynæcology. It approaches the subject from the clinical and practical standpoint, and is stamped throughout with the author's personality. Lewers' "Practical Text-book of the Diseases of Women" (Lewis, 10s. 6d.) is a smaller work, popular with students, but less suited to the practitioner. Galabin's "Diseases of Women" (Churchill, 16s. net) is a well-written, useful text-book of gynæcology for students and practitioners. Jellett's "Short Practice of Midwifery" (Churchill, 10s. 6d. net) is a very sound and succinct account of the subject with which it deals.

---

"Green's Pathology" (1 vol., Renshaw, 18s. net, 10th edition, by W. C. Bosanquet). A reliable introduction to pathology, well illustrated, readable, and up to date. "Martin's Pathology" (15s. net) is also an excellent manual; while Bland-Sutton's "Tumours" (Cassell, 21s.) is an interesting book which all students should read.

Muir and Ritchie's "Manual of Bacteriology" (Henry Frowde and Hodder and Stoughton, 10s. 6d. net). Perhaps the most useful work of its kind. A good introduction to the science of bacteriology.

Emery's "Clinical Bacteriology and Hæmatology" (Lewis, 7s. 6d.) is specially written for practitioners.

Hutchison and Rainey's "Clinical Methods" (Cassell and Co., 10s. 6d.). Almost indispensable to the clinical clerk and the scientific physician. In connection with the subject of clinical methods Dr. Gee's "Auscultation and Percussion" (Oxford Medical Publications, 5s. net) should be mentioned. It is a book which every senior student should procure and keep with him throughout his professional career.

Among useful works on Hygiene and Public Health are Whitelegge and Newman's "Hygiene and Public Health" (Cassell, 7s. 6d.), Hamer's "Manual of Hygiene" (Churchill, 12s. 6d.), Parkes and Kenwood's "Hygiene and Public Health" (12s.), and Ham's "Handbook of Sanitary Law" (2s. 6d.).

The most generally useful and popular handbooks on Forensic Medicine are Dixon Mann's "Forensic Medicine and Toxicology" (21s.), Husband's "Forensic Medicine, Toxicology, and Public Health" (edited by Buchanan and Hope, 10s. 6d. net), and Taylor's large "Principles and Practice of Medical Jurisprudence" (revised by F. J. Smith, 36s. net).

---

*Diseases of Children.*—Among medical works those of Goodhart and Still (Churchill, 12s. 6d.), Eustace Smith (W. Green and Sons, 21s.), and Cautley (Churchill, 7s. 6d.), should be mentioned.

Ashby and Wright's "Diseases of Children" (Longmans, Green, 21s. net) is convenient because it contains the medical and surgical diseases in a single volume.

Edmund Owen's "Surgical Diseases of Children" (Cassell, 21s.) and D'Arcy Power's "Surgical Diseases of Children and their Treatment by Modern Methods" (Lewis, 10s. 6d.) are well illustrated, and give the views of experienced surgeons.

*Nervous Diseases.*—Sir Wm. Gowers' large work (Churchill, 35s.) is the standard authority on this subject. Clutterbuck's "Nerve Diseases" (Scientific Press, Ltd., 3s. net) will be found of service. Beevor's "Diseases of the Nervous System" (Lewis, 10s. 6d.) is well adapted for the final examinations.

*Insanity.*—The manuals of Savage (Cassell, 9s.), Craig, and Clouston (Churchill, 14s.) are the most popular, and will be found sufficient.

